# ‘I Don't Think There Is a One‐Size‐Fits‐All’: A Qualitative Study Exploring Healthcare Professional and Service Provider Perspectives of Using Innovative Models of Cervical Screening to Improve Equitable Access to Self‐Collection

**DOI:** 10.1002/cam4.70981

**Published:** 2025-05-25

**Authors:** Claire Bavor, Tessa Saunders, Mikayla Wolfe, Megan A. Smith, Nicola Creagh, Deborah Bateson, Angela Kelly‐Hanku, Paula Jops, Marion Saville, Natalie Taylor, Kate Broun, Julia M. L. Brotherton, Claire Nightingale

**Affiliations:** ^1^ Evaluation and Implementation Science Unit, Centre for Health Policy, Melbourne School of Population and Global Health University of Melbourne Melbourne Victoria Australia; ^2^ The Daffodil Centre University of Sydney, a Joint Venture With Cancer Council NSW Sydney New South Wales Australia; ^3^ Sydney Medical School, Faculty of Medicine and Health The University of Sydney Sydney New South Wales Australia; ^4^ Kirby Institute University of New South Wales Sydney New South Wales Australia; ^5^ Australian Centre for the Prevention of Cervical Cancer Carlton Victoria Australia; ^6^ Implementation to Impact School of Population Health UNSW Sydney Kensington New South Wales Australia; ^7^ Cancer Council Victoria Melbourne Victoria Australia

**Keywords:** cervical screening, HPV, models of screening, self‐collection, self‐sampling, self‐testing

## Abstract

**Introduction:**

In the Australian National Cervical Screening Program (NCSP), self‐collection can be performed in any setting deemed appropriate by the healthcare professional who orders the test, creating opportunities to develop innovative cervical screening models that can address known barriers to access for under‐ and never‐screened women and people with a cervix. This study explored the acceptability and appropriateness of innovative models and key considerations for their design and implementation from the perspectives of clinical and non‐clinical providers.

**Methods:**

We conducted online, semi‐structured interviews with healthcare professionals, pathology providers and community service providers (June–October 2023). Data were analyzed using template analysis, a form of thematic analysis.

**Results:**

There were 132 participants from across Australia (82 clinical providers [e.g., doctors, nurses, midwives]; 34 non‐clinical providers [e.g., health/community service staff, disability support workers, bicultural workers]; and 16 pathology sector professionals). Four overarching themes were identified: acceptability, appropriateness, screening quality and safety, and implementation considerations. Most found innovative models acceptable when appropriately tailored to the needs of different population groups, particularly through community outreach, home in‐reach and peer‐supported services. Embedding clinical governance and oversight in the cervical screening pathway was a high priority to ensure that screening participants received adequate information about cervical screening and appropriate follow‐up care. Participants identified the need for clearly defined roles in the cervical screening pathway, sustainable funding and professional development opportunities to expand the role of nurses and optimize the roles of non‐clinical providers.

**Conclusions:**

Innovative models of cervical screening using self‐collection can offer more accessible, inclusive, and convenient care, especially for under‐ and never‐screened populations. Clinical governance and oversight must be embedded in the cervical screening pathway to maintain high‐quality screening services and to support the implementation of tailored and targeted innovative screening models.

## 1 Introduction

Human papillomavirus (HPV) testing is the most effective way of screening to prevent cervical cancer and is recommended by the World Health Organization for use in all settings [1, 2, 3]. A major advantage of HPV screening is that the test can be performed equally well on a lower vaginal sample collected by the screening participant using a flocked swab (‘self‐collection’) or a cervical sample collected by a healthcare professional using a speculum (‘clinician‐collection’). As there is flexibility in where self‐collection can be performed, it offers greater autonomy, privacy and convenience for screening participants and is widely accepted by screen‐eligible people and healthcare professionals [4, 5]. Numerous trials show that self‐collection increases screening uptake among under‐ and never‐screened population groups, regardless of whether self‐collection is offered in a clinic, the community or a person's home [6]. This flexibility has been explored globally using community outreach, mail‐to‐all and opt‐in screening models [6]. Some high‐income countries, including the Netherlands, Denmark and Sweden, have integrated this flexibility into their national HPV‐based screening programmes, enabling screening participants to opt‐in to receive self‐collection kits by mail [7, 8, 9]. Australia does not currently have a centralised mail‐out option for self‐collection, as the National Cervical Screening Program (NCSP) is embedded in primary care. In the NCSP, screening participants receive a routine, written screening invitation/reminder that includes information about self‐collection every 5 years from the National Cancer Screening Register. They may also be routinely invited by their regular primary care provider or be invited opportunistically at a primary care service.

To achieve the equitable elimination of cervical cancer as a public health problem (< 4 cases per 100,000), Australia must address persistent disparities in the incidence of cervical cancer (6.6 per 100,00 people in 2020) [3, 10]. To improve equity in the NCSP, screening guidelines since July 2022 have recommended that all women and people with a cervix aged 25–74 be offered five‐yearly HPV testing on their choice of a clinician‐collected or self‐collected sample [11, 12]. Only medical or nurse practitioners with a Medicare provider number can order a cervical screening test and receive reimbursement under Medicare, Australia's universal healthcare insurance scheme. This means that cervical screening, including self‐collection, is primarily accessed via primary care, where eligible screening participants obtain and return the self‐collection kit to complete at the clinic or in any setting deemed appropriate by the healthcare provider [1]. Screening tests are funded by Medicare for eligible people (aged 24 years 9 months or older, with no test in the preceding 57 months); however, some may incur an out‐of‐pocket fee from the pathology provider if they charge at a rate higher than the government reimbursement rate or from the healthcare professional if the consultation is not fully covered by the service's Medicare rebate (i.e., it is not ‘bulk billed’). Currently, 42.6% of the Australian population lives in a region without access to a bulk billing only practice, and an increasing number of Australians are delaying visits to primary care due to rising costs, limited availability and long wait times, especially in lower socioeconomic and rural areas [13, 14, 15]. For some population groups, these challenges are compounded by barriers to accessing cervical screening, including limited access to culturally safe, inclusive and accessible health services and providers [16, 17, 18], cultural and religious beliefs [19], and a lack of knowledge of cervical cancer and its prevention [16, 19].

To help address some of these barriers to accessing screening, the NCSP guidelines offer flexibility in the settings where self‐collection can be performed and the types of healthcare professionals involved, as long as the cervical screening test is ordered and overseen by a healthcare professional [11]. This enables innovative models of cervical screening to be developed that may better meet the needs of different population groups. For example, self‐collection may be offered via different forms of community outreach (e.g., community events, home in‐reach, mobile screening buses) and in a broader range of clinical settings (e.g., antenatal care, peer‐supported services and telehealth) [11]. There is limited evidence on the effectiveness of these models in Australia; although various community outreach models have been trialled internationally [6] alongside other novel models such as telehealth [20] online ordering after an appointment [21] integrated breast, bowel and cervical screening [22] and pharmacy pick‐up [23]. There is, however, local evidence that similar models improve access to testing for sexually transmitted infections (STIs) [24].

The *National Strategy for the Elimination of Cervical Cancer in Australia* (Australian Elimination Strategy) is equity‐focused and includes a key strategic priority to expand by who, where and how cervical screening is offered to improve its reach and uptake [25]. To ensure that self‐collection reaches those who it may benefit the most, evidence is needed to inform the implementation of innovative cervical screening models within the NCSP. We aimed to explore healthcare professionals' and service providers' perspectives on the acceptability of innovative models of cervical screening to increase equity in the Australian NCSP, alongside barriers and enablers to their implementation.

## 2 Methods

This qualitative study is part of a national project, *Supporting Choice for Cervical Screening*, which aims to generate evidence about how the choice of self‐collection can be implemented to improve equitable access to cervical screening [25].

We invited healthcare professionals, pathology sector professionals and community service providers who work in services for the general public or for any of the three specified priority population groups to participate in a semi‐structured interview about how the choice of self‐collection had been implemented in their service and what is needed to promote participation among the population group/s they serve. The three priority population groups were women and people with a cervix who (1) are from refugee and asylum seeker backgrounds; (2) have physical or sensory disability; and/or (3) identify as part of the LGBTQI+ community. These population groups were selected as they experience specific barriers to accessing screening [16, 17, 18, 25]. Perspectives from members of these communities have been explored in *Supporting Choice for Cervical Screening* but will be analysed and reported separately. The findings reported here focus on healthcare professional and service provider perspectives relating to innovative screening models.

### 2.1 Participants and Recruitment

Participants were recruited from across Australia to capture perspectives from those working in different settings, roles and locations. Participants were eligible if they were working in a clinical role (e.g., doctors, nurses, midwives), a non‐clinical role (e.g., programme staff, managers) or in the pathology sector. Table 1 defines participant roles in cervical screening as used for this study.

**TABLE 1:** Definition of participant roles in cervical screening as defined for this study.

Role		Role in cervical screening
Clinical healthcare professionals (screening providers)	Doctors (medical practitioners including: general practitioners [GPs], GP registrars, physicians/specialists)	A healthcare professional who can order and oversee a cervical screening test. Holds a Medicare provider number and can sign the pathology request form so the test is reimbursed by Medicare.
	Nurse practitioners	A healthcare professional who can order and oversee a cervical screening test. Holds a Medicare provider number and can sign the pathology request form so the test is reimbursed by Medicare.
	Nurses^a^ (includes: practice nurses, sexual health nurses, clinical nurse specialists)	Nurses can offer cervical screening, but tests are only reimbursable under Medicare if the pathology request form is signed by a doctor or nurse practitioner.
	Midwives^a^	Midwives can offer cervical screening, but tests are only reimbursable through Medicare if the pathology request form is signed by a doctor or nurse practitioner.
Non‐clinical service providers	Peer workers, bicultural workers, disability support workers	Peer‐ and bicultural workers and disability support workers may promote and support participation in screening at health services that provide cervical screening.
	Manager/supervisors (includes: practice/service/programme managers, directors and coordinators)	Managers and supervisors may work in organisations that provide cervical screening (i.e., sexual and reproductive health services) and/or oversee a service/programme of work that can promote participation in screening and/or support its clients to access cervical screening.
	Programme staff (includes: training coordinators, health promotion officers, counsellors, GP Liaison officers)	Programme staff may work in organisations that provide cervical screening (i.e., sexual and reproductive health service) and/or at a service that can promote participation in screening and/or support its clients to access cervical screening.
Pathology sector professionals	Pathology sector professionals (includes: pathologists, molecular biologists, cytopathologists)	Pathology sector professionals support the delivery of the NCSP through their professional interpretation of, and reporting on, screening and follow‐up results and by conducting and overseeing the laboratory processing of samples and supply of consumables.

^a^ Nurses and midwives are encouraged to undergo training and credentialing before offering cervical screening; however, this is not mandatory.

Participants were predominately identified through the study team's extensive network, including its Provider and Implementation Advisory Group (16 healthcare and policy professionals) and Community and Consumer Advisory Panel (12 women from the community), and emailed a personally addressed invitation. The study was also promoted on the social media accounts and newsletters of partner organisations, peak bodies and Primary Health Networks. Prospective participants completed an expression of interest form and were emailed further information about the study. Snowball sampling was used to recruit additional participants [26]. All eligible participants were sent a copy of the plain language statement and consent form.

### 2.2 Materials

The research team (C.B., T.S., M.W., M.C., N.C., K.F., S.R., C.N.) developed an interview guide for each of the three participant groups that were tailored to their role in cervical screening (Table 2). All participants were asked about how their service offered self‐collection (data to be reported elsewhere). Participants were also asked what they thought about innovative cervical screening models, including their acceptability, advantages, disadvantages and what is needed to support their implementation. Interview guides were reviewed by the study's Provider and Implementation Advisory Group. The interview questions are provided in the Supporting Information.

**TABLE 2:** Overview of interview guides developed for each participant group.

Interview guide	Population group served	Study participant role	Service type
1	General population	Clinical: healthcare professionals	Primary and tertiary health services (e.g., primary healthcare services, sexual and reproductive healthcare services, public hospitals)
2	Women and people with a cervix who are from refugee and asylum seeker backgrounds, have a physical or sensory disability and/or identify as part of the LGBTQI+ community	Clinical: healthcare professional Non‐clinical: service providers	Health and community services
3	General population	Pathology sector professionals (e.g., pathologists, laboratory managers)	Public and private pathology laboratories

### 2.3 Data Collection

Semi‐structured interviews were conducted online over Zoom with one or two research team members (C.B., T.S., M.W., M.C., N.C., K.F., S.R., C.N.) who are women and experienced in conducting qualitative interviews. Interviews were conducted individually or as a group with colleagues if preferred. All interviewers followed the appropriate interview guide using prompts to gather further details and clarification. Participants discussed the innovative models they found most relevant to their role.

All interviews were audio‐recorded and transcribed verbatim using Otter.ai. Transcripts were deidentified and cleaned by the research team to ensure accuracy and completeness. Participants could request to review a copy of their transcript.

### 2.4 Data Analysis

The interview data were analysed using template analysis; a form of thematic analysis that uses a coding framework to hierarchically organise meaningful themes in the data [27]. This method was chosen to allow for the perspectives of different groups in the study to be examined and compared [28]. After reading all transcripts, one author (C.B.), a PhD student with formal training in qualitative research, developed a coding framework using a set of a priori themes based on the study aim [28]. The coding framework was reviewed by the research team (C.N., T.S.) and then applied to all the data by one author (C.B.). It was consistently revised and refined in collaboration with the research team (C.N., T.S.) to ensure it continued to reflect the data. Codes corresponding to each model of screening discussed were used as ‘placeholders’ to organise data under different themes to enable comparison of codes across models. Most revisions to the framework were made to capture specific advantages, disadvantages and implementation considerations under each theme [28]. The final coding framework is available in the Supporting Information. In the framework, acceptability was defined as healthcare professionals' satisfaction with innovative models and appropriateness was defined as the perceived fit and relevance for different population groups [29]. Data were analysed using NVivo (release 1.6.1, QSR International Pty Ltd).

### 2.5 Ethics

This study received ethics approval from the University of Melbourne Human Research Ethics Committee (2023‐26114‐39203‐4). Additional ethics approval was received from two community organisations that provide services and advocacy for LGBTQI+ people, ACON (202316) and Thorne Harbour (TTH/CREP/23/006). All participants provided written, informed consent before the interview. Those participating in an interview outside of their salaried time were offered a $100 AUD gift card.

## 3 Results

One hundred individual and group interviews were conducted with 132 participants from across Australia between June and October 2023 (Table 3). The average length of an interview was 47 min (range: 24–78 min). Eighty‐two participants had a clinical role as a doctor, nurse practitioner, nurse or midwife. Of these, 29 were GPs/physicians, 23 were nurses/midwives providing cervical screening autonomously, 19 were nurses or midwives who were currently conducting cervical screening but did not have a Medicare provider number, and 11 were nurses and midwives who would like to offer cervical screening but did not at the time of the interview. A total of 34 participants had a non‐clinical role in a health or community service for a selected priority population. Sixteen participants worked in a pathology service.

**TABLE 3:** Participant demographics and roles (*N* = 132).

Characteristic		*N* (%)
Gender	Woman	73 (55.3)
	Man	4 (3.0)
	Non‐binary and other^a^	3 (2.3)
	Missing^b^	52 (39.4)
State	New South Wales	34 (25.8)
	Victoria	32 (24.2)
	Queensland	19 (14.4)
	South Australia	16 (12.1)
	Western Australia	12 (9.1)
	Australian Capital Territory	8 (6.1)
	Tasmania	8 (6.1)
	Northern Territory	3 (2.3)
Clinical providers	GPs/physicians	29 (22.0)
	Nurses and midwives who provide autonomous cervical screening (nurse practitioners, women's health nurses with specific funding schemes)	23 (15.2)
	Nurses and midwives with cervical screening provider training	19 (14.4)
	Nurses and midwives without cervical screening provider training	11 (8.3)
Non‐clinical providers	Manager/supervisor	18 (13.6)
	Programme staff	11 (8.3)
	Other^c^	5 (3.8)
Pathology providers	Pathology sector professionals	16 (12.1)
Population served	Health service for the general public (including people from priority groups)	58 (43.9)
	Health or community service for refugee and asylum seeker backgrounds	25 (18.9)
	Health or community service for people with disability	22 (16.7)
	Health or community service for the LGBTQ+ community	11 (8.3)

^a^ Non‐binary and other category includes self‐reported terms participants used to describe their gender.

^b^ Gender data is missing for 52 participants as it was not collected initially.

^c^ Other category includes bicultural workers, peer workers, counselors and GP liaison officers.

Fifty‐eight participants worked in a general practice, sexual and reproductive health service or state/territory‐funded health service that provided care to the general public (53 clinical; 5 non‐clinical). There were another 58 participants from health and community services specifically for the selected priority populations, including 25 participants from refugee and asylum seeker services (18 clinical; 7 non‐clinical); 22 from disability services (7 clinical, 15 non‐clinical); and 11 from LGBTQI+ services (4 clinical; 7 non‐clinical).

### 3.1 Themes

Four overarching themes were identified relating to innovative screening models: acceptability; appropriateness; maintaining screening quality and safety; and implementation considerations.

### 3.2 Acceptability of Innovative Models of Cervical Screening

Most clinical providers and all non‐clinical providers agreed that implementing innovative models to improve access to self‐collection for priority population groups was acceptable. Many participants discussed how expanding where self‐collection was offered would enable opportunistic screening in different settings and better support the population groups they worked with:

If they're not coming to us as GPs, then we need to find ways that we can encourage women to find another way of getting their screening done. (P96, GP, general population)

Most study participants felt that the benefits of implementing innovative models to improve screening participation outweighed the disadvantages. However, for clinical providers, acceptability was often linked to their role in providing high‐quality and holistic care. Many valued cervical screening as an important opportunity for a wider health check, notably sexual, gynaecological and pelvic floor health:

You can use your general consults as an opportunistic situation for getting your cervical screening test done. But likewise, someone coming and talking to you about their screen… can be an opportunistic pick up of other problems. (P98, GP, general population)

Despite the wide acceptability, a few clinical and pathology providers did not think innovative models were at all acceptable or were more hesitant. They had concerns about the quality of the sample outside of a clinical setting and the potential need to return for a clinician‐collected test. Therefore, they thought innovative models were only acceptable for targeting under‐screened population groups:

I don't really see a need for expanding [self‐collection] further than what it is at the moment unless it is some kind of specific home collection thing for the remaining people not participating. (P66, pathology provider)

### 3.3 Appropriateness of Innovative Models of Cervical Screening

Participants found some screening models to be more appropriate than others for the population group they worked with:

I don't think there is a one size fits all. (P123, GP, general population)

Those who worked with priority population groups discussed the challenges their clients face accessing inclusive, culturally appropriate and accessible health services, so accessing self‐collection outside of a clinical setting could be more private, comfortable and safe. As described in Table 4, these participants identified screening models that could be appropriate for the population group they worked with, especially if developed in partnership with community members and organisations.

**TABLE 4:** Examples of how innovative screening models may address barriers to screening experienced by different population groups.

Appropriate screening models for different population groups	How the model supports participation in screening for the population group	Participant quote
Community outreach/events for people from refugee and asylum seeker backgrounds	Delivered in partnership with bicultural workers Culturally safe Delivered in language Addresses medical fear and stigma	‘A bicultural worker could run a private session and provide them with what they need to do self‐collection… no one has to take anything anywhere, they can give it back to the nurse… that would help address power imbalances, they'd be in a familiar space, in a comfortable environment and they'd have peer support around’. (P4, nurse, non‐screening provider, refugee & asylum seeker service)
Home in‐reach for people with disability	Overcomes logistical barriers to organising appointment (e.g., transport, accessible service) Safe, private space	‘I think that there are ways it can be done considerably and sensitively… because the transport element would be enough of a logistical barrier for support organisations…who forget about sexual health testing’. (P48, manager/supervisor, disability support organisation)
Community events and peer‐based services for the LGBTQ+ community	Supportive and trauma‐informed care Inclusive Opportunistic	‘The emotional energy of navigating inappropriate or uninformed health providers … you have higher rates of trauma… if you don't have a GP who understands that… it's just layers of things I'd say’. (P85, manager/supervisor, LGBTQ+ advocacy organisation)
Telehealth, mobile screening buses, pharmacy pick‐up, mail‐to‐all for the general population	Low or no cost to access for the participant Overcomes challenges to accessing GPs in rural areas Convenient	‘There are so many people who don't have a GP or can't get a GP appointment… access to care is the barrier for a lot of people. And I guess there has to be a solution to that problem’. (P118, GP, general population)

However, many participants thought certain screening models would not be appropriate for the priority population group they work with as they could *add* barriers to access. For example, participants working with people from refugee and asylum seeker backgrounds identified barriers to participating via mail and telehealth models, including a lack of a permanent address, language, limited access to a telephone and/or Internet and lack of knowledge or awareness of cervical screening:

I think a mail‐out for newly arrived refugees may not be too beneficial. Main reason being they need a lot of support… mail‐out wouldn't necessarily be a priority of theirs, their health concerns are right here, right now. (P14, nurse, non‐screening provider, refugee & asylum seeker service)

### 3.4 Maintaining Cervical Screening Quality and Safety

The perceived advantages and disadvantages of the different models offered outside of a clinical setting were often linked to whether the respondent thought that screening participants could be provided with adequate information about screening. Clinical providers were particularly focused on ensuring screening participants had enough information to make an informed decision about participating in screening. Moreover, they thought this discussion was critical for history taking and ensuring they were asymptomatic and therefore eligible to participate in screening:

[The cervical screening provider] also must know who is not eligible. Some people actually really want self‐screening, but they shouldn't have it. They're presenting with abnormal bleeding, had post‐coital bleeding, or they have a recent abnormal cervical screen, and they're in the process of follow‐up… all those things that they may not reveal openly unless they're asked. (P13, GP, LGBTQI+ service)

Subsequently, clinical providers thought screening models where the screening participant could have this discussion in a private space were more acceptable than pharmacy and community‐based models. Many participants also expressed these concerns about mail‐out models, with the added worry that, without appropriate support, the test would be forgotten about:

[The self‐collection kit is] posted to you, so it's very easy to ignore. But if you're sitting in front of a doctor or nurse or somebody offering it to you, you're more likely to do it…. You can't just go, ‘Yeah, whatever,’ and chuck it out. (P61, nurse cervical screening provider, LGBTQI+ service)

A principal concern discussed across all models was ensuring screening participants were supported to access follow‐up care, including clinician‐collection of a cervical sample or colposcopy, if required. Some clinical providers highlighted that the success of cervical screening goes beyond the initial screening test; and that the provision of screening results needs to be carefully considered to ensure screening participants who may be confused or anxious about the results are well supported:

It was her first cervical screening test, I went through exactly what the tests meant, what I was looking for… I rang her up to give her non‐16/18 [liquid‐based cytology] negative, and she just absolutely freaked…so where's the support for those people? (P117, nurse cervical screening provider, general population)

### 3.5 Implementation Considerations

To implement innovative screening models safely and appropriately, clinical providers identified key considerations for the cervical screening pathway. Participants highlighted the importance of clinical governance and oversight, and clearly defined processes, roles and responsibilities of clinical, non‐clinical and pathology providers, health services and the NCSP (Table 5):

As long as there's a nice, clear clinical pathway. (P24, pathology provider)

**TABLE 5:** Key considerations for implementing safe and appropriate cervical screening.

Questions at different steps in the cervical screening pathway	Participant quote
1. How is eligibility determined?	‘How do we manage those people that don't have a normal screening history?’ (P16, nurse cervical screening provider, general population)
2. Who provides the self‐collection kit and how? What happens if it's not returned?	‘How do we get the package to them [following a telehealth consultation]? Do they come in and pick it up?… Is it our jobs as clinicians to track them down if they haven't done it?’ (P129, nurse cervical screening provider, general population)
3. When is the self‐collection kit returned and how?	‘[The screening participant has] got to think about – “oh, how soon should I get my swab back to my GP? Or how do I send it back?”… I wonder whether those samples can survive that trip?’ (P123, GP, general population)
4. Which pathology provider tests the swab? What is the cost?	‘Where the [swab] is going to get sent, which provider? Are they going to go all over the place to different pathology labs, or are they going to [our usual laboratory]?’ (P40, nurse cervical screening provider, general population)
5. Who is responsible for providing the results and linkage to follow‐up care?	‘Who's managing the results? Who's managing the abnormal results? What happens with recall? Who gets hold of patients? Who is linking to care? Who's responsible for the subsequent testing?’ (P95, GP, general population)
6. Who provides follow‐up care and how?	‘Coming back for follow‐up if [the cervical screening test's] done through the chemist or community health? Are they aware of who to go to? If they don't have a regular GP, what happens?’ (P97, GP, general population)

### 3.6 Resources Required to Support Implementation

Participants identified ways that the NCSP could support health services and healthcare providers to implement innovative models. Many thought that increasing the scope of practice for nurses, especially practice nurses and midwives, to order and oversee cervical screening without a GP would be critical:

In theory, it gives you lots more flexibility but in practice because of the way the service is funded in Australia, the fact you still need a doctor, it just really holds us back in terms of what we can do. (P11, non‐clinical manager, general population)

Clinical and non‐clinical providers wanted further clarification about the role that non‐clinical providers, such as peer‐workers, bicultural workers and disability support workers, could play in supporting access to self‐collection. A few clinical providers were concerned about the scope of non‐clinical providers in self‐collection without clinical training:

It's like this kind of balancing act of having access, but also having the right people providing the services. Because it's rare that, you know, you're not asked other questions, or there's not other things. (P118, GP, general population)

However, many advocated for professional development opportunities to promote the role of non‐clinical providers in supporting access to screening with a cervical screening provider:

I think our non‐clinical workers are extremely underutilized and undervalued… They're the ones the community is going to trust. If I go in and then say, ‘Oh you need to do this,’ they'll say whatever. If [a trusted non‐clinical provider] goes in and tells them, they'll do it. (P110, midwife cervical screening provider, general population)

Most participants would need sustainable funding to support the staffing, logistical and administrative costs associated with implementing new screening models. This included support for nurses and midwives to access accredited cervical screening provider courses:

It's the time that nurses or clinicians are taken away from the clinic, so it's just paying for their time to do that. (P15, nurse cervical screening provider, refugee & asylum seeker service)

Finally, participants discussed the value of partnering with trusted clinical and community services to deliver innovative models linked to clinical care. Likewise, they emphasised the importance of working with community members and using co‐design to develop appropriate screening models.

You know, the people in their communities know best, so we need to work with them. (P46, nurse screening provider, general population)

## 4 Discussion

This study explored the perspectives of healthcare professionals and service providers on designing and implementing innovative models to safely and appropriately increase equitable access to self‐collection. We found that, as long as providers were reassured that appropriate clinical governance and oversight could be achieved, they supported using the appropriate innovative model/s to provide culturally safe, accessible and inclusive care for population groups more likely to be under‐ and never‐screened. Our findings highlight the key considerations for implementing safe cervical screening pathways, including for follow‐up care. The findings can be used to inform the implementation of targeted and tailored screening models to improve the reach of self‐collection, in line with the strategic priorities outlined in the Australian Elimination Strategy [25].

Previous research has found that self‐collection improves access to screening for under‐ and never‐screened participants regardless of how or where it is offered [6]. Data from the NCSP further demonstrates self‐collection uptake is higher among under‐screened people and population groups that have tended to be under‐screened [30]. Our findings build upon this evidence base by highlighting the wide acceptability of innovative models among healthcare professionals for addressing existing barriers to accessing primary care in Australia and providing greater convenience. However, as there is no one‐size‐fits‐all approach, the implementation of innovative models within the NCSP must account for the needs of different population groups. For example, clinical providers most often discussed mail‐out, telehealth and pharmacy pick‐up models as convenient and low‐cost ways of expanding access to self‐collection for the general population. While these models address important systemic barriers to accessing primary care, their appropriateness for different population groups and who they may and may not support to access cervical screening must be considered. For example, providers felt that culturally and linguistically diverse people are less likely to engage with telehealth, and this is supported by other research [31]. Community outreach, with involvement from bicultural workers and home in‐reach, was suggested as alternative models to engage specific population groups. Australia's National Bowel Cancer Screening Program has recently responded to a need for increased flexibility in their model of screening, now enabling healthcare professionals to directly issue screen‐eligible people in the clinic as a supplement to the mail‐out approach. This demonstrates how population‐based programmes can adapt to better reach priority population groups [32, 33].

Trusted healthcare professionals, such as GPs and nurses, play an important role in supporting under‐ and never‐screened participants to complete the cervical screening pathway [34]. This is one of the key reasons why self‐collection has largely remained embedded in primary care in Australia. In our study, clinical providers emphasised the need to support screening participants in accessing any required clinical services after the initial screening test, especially for models facilitated outside of primary healthcare (e.g., community outreach). Data from the Australian NCSP indicates that colposcopy attendance rates are similar among people using self‐collection and those screening via clinician‐collection within 6 months (81.0% vs. 80.4%) [30]. Similarly, 82.7% of those who needed a clinician‐collected sample for cytology following HPV detection on a self‐collected sample attended within 6 months [30]. This is consistent with high follow‐up rates reported in a trial in England that offered self‐collection to people overdue for screening (84.9%) [35], and very high rates in the Netherlands (93%) [36]. However, some participants may need additional support to ensure timely access to follow‐up services, with a recent meta‐analysis finding that follow‐up rates across screening models are highly variable, ranging from 41.0% to 100.0% (noting considerable heterogeneity in study design) [6]. High follow‐up rates for mail‐out models have been achieved by pre‐booking triage appointments with GPs and gynaecologists [37], and also by sending results to both screening participants and their GPs [38]. Participants in our study further identified the need for robust referral pathways and strong partnerships with clinical and community services to address barriers to follow‐up care for all models.

In our study, clinical providers reiterated their role in providing safe and appropriate cervical screening, and thus identified embedding clinical safety and quality into different cervical screening pathways as a high priority. Guidance for implementing safe, high‐quality care for self‐collection should be aligned with the NCSP quality framework [39], and draw on previous experience and evidence. For example, self‐collection is extensively used to increase the reach of testing services for various STIs, including chlamydia, gonorrhoea and trichomoniasis [24]. The process is similar for cervical screening, whereby the self‐collected sample is sent by the healthcare professional to the laboratory for testing, and positive results require clinical care [40]. Community outreach [41], online ordering [42] and mail‐to‐all [43] models have been shown to increase uptake of testing for these STIs for priority population groups; as have outreach and community‐ and peer‐led models for HIV rapid testing [44], and point‐of‐care testing for hepatitis B and C [45]. One study that compared the effectiveness of hepatitis B screening and linkage to care in clinical settings to non‐clinical settings showed similar levels of follow‐up in both settings could be facilitated through strong partnerships between community‐based organisations, community healthcare workers and doctors [46]. The importance of these partnerships was emphasised by study participants when considering the design and implementation of innovative models.

Implementation guidance and support are required from the government, pathology providers and health services to realise the potential of self‐collection within the NCSP and ensure safety and quality are maintained. The flexible delivery of Australia's NCSP is currently limited by inflexible funding models and Medicare requirements, which largely preclude the sustainable involvement of many healthcare providers in delivering flexible screening models. Many nurses in this study who were cervical screening providers felt that their role in screening was limited due to being unable to order a cervical screening test under Medicare without a GP signature. There are calls for more flexible funding models that enable nurses to work to their full scope of practice and independently request and order cervical screening [25, 47]. Additionally, many participants supported professional development for peer, bicultural and disability support workers to enable them to promote participation in cervical screening. Finally, cervical screening providers need further guidance from pathology providers about the stability of samples collected outside of a clinical setting during the period they are in transit to the laboratory.

The strengths of this study include purposively interviewing a diverse group of healthcare professionals to capture the clinical perspectives of those providing cervical screening and insights from providers working directly with community members from the priority population groups. However, the number of participants from some jurisdictions was limited, as was the number working with people from the LGBTQI+ community. A parallel body of research working to improve the delivery of screening for Aboriginal and Torres Strait Islander people should also be noted. There may have also been a response bias towards those who have adopted and/or are supportive of self‐collection. Furthermore, while interview guides were tailored to the participant group to ensure relevant information was collected, participants only discussed the screening models they perceived to be the most interesting or relevant to their setting, meaning some screening models were discussed more than others.

This study has important implications for policy and practice. It highlights how the multi‐faceted nature of equity needs to be considered when implementing innovative screening models to avoid unintentionally introducing barriers. We identified important considerations for optimising access to self‐collection within the NCSP, presenting findings that can inform the implementation of the Australian Elimination Strategy, which strives for the equitable elimination of cervical cancer as a public health problem [25]. Globally, the number of countries recommending HPV‐based screening is increasing, including those offering self‐collection [36]. Countries can learn from each other to ensure that priority population groups can access high‐quality and safe screening in ways that suit them. Further research to better understand how screening models can be adapted for different population groups would build the implementation evidence base needed to attain sustainable funding sources and allow scale‐up. Finally, research with community members is needed to ensure the voices of those in priority population groups are heard and is underway as part of *Supporting Choice for Cervical Screening*.

## 5 Conclusion

Our study demonstrates that innovative models of cervical screening using self‐collection are broadly acceptable to healthcare professionals and service providers, especially when tailored to the needs of under‐ and never‐screened population groups. Self‐collection offers flexibility that can be leveraged to overcome barriers to screening, such as through community outreach, home in‐reach and peer‐supported models. To ensure clinical safety and effectiveness, implementation guidance is needed to outline procedures in the cervical screening pathway, including eligibility assessment, results management and follow‐up care. Expanding the role of nurses and non‐clinical providers through professional development opportunities and sustainable funding models will also be essential for successful implementation. Strong partnerships with community organisations and the co‐design of services with priority population groups will be critical to the development of tailored, targeted and sustainable screening models. These findings offer valuable insights for strengthening the Australian NCSP and supporting progress towards the equitable elimination of cervical cancer as a public health problem.

## Author Contributions

**Claire Bavor:** conceptualisation, methodology, investigation, formal analysis (lead), project administration, writing – original draft. **Tessa Saunders:** conceptualisation, methodology, investigation, validation, formal analysis, supervision, project administration, writing – reviewing and editing. **Mikayla Wolfe:** methodology, investigation, writing – reviewing and editing. **Megan A. Smith:** conceptualisation, methodology, investigation, supervision, funding acquisition, writing – reviewing and editing. **Nicola Creagh:** conceptualisation, investigation, funding acquisition, project administration, writing – reviewing and editing. **Deborah Bateson:** conceptualisation, funding acquisition, writing – reviewing and editing. **Angela Kelly‐Hanku:** conceptualisation, funding acquisition, writing – reviewing and editing. **Paula Jops:** writing – reviewing and editing. **Marion Saville:** funding acquisition, writing – reviewing and editing. **Natalie Taylor:** funding acquisition, writing – reviewing and editing. **Kate Broun:** funding acquisition, writing – reviewing and editing. **Julia M. L. Brotherton:** conceptualisation, methodology, funding acquisition, writing – reviewing and editing. **Claire Nightingale:** conceptualisation, methodology, investigation, validation, formal analysis, supervision, funding acquisition, project administration, writing – reviewing and editing.

## Ethics Statement

This study received ethics approval from the University of Melbourne Human Research Ethics Committee (2023‐26114‐39203‐4). Additional ethics approval was received from two community organisations that provide services and advocacy for LGBTQI+ people, ACON (202316) and Thorne Harbour (TTH/CREP/23/006). All methods were carried out per the relevant guidelines. All participants provided written, informed consent before the interview. Those participating in an interview outside of their salaried time were offered a $100 AUD gift card.

## Conflicts of Interest

M.S. is an employee of the Australian Centre for the Prevention of Cervical Cancer (ACPCC) which has received donated laboratory tests, equipment and funding for HPV validation studies and self‐collection related research from multiple manufacturers. J.M.L.B. is a former (within last 3 years) employee of the Australian Centre for the Prevention of Cervical Cancer (ACPCC) which has received donated laboratory tests, equipment and funding for HPV validation studies and self‐collection related research from multiple manufacturers.

## Supporting information


Data S1.

## Data Availability

Datasets used and/or analyzed during the current study are available from the corresponding author on reasonable request.
