# Supplementary material for: ‘I Don't Think There Is a One‐Size‐Fits‐All’: A Qualitative Study Exploring Healthcare Professional and Service Provider Perspectives of Using Innovative Models of Cervical Screening to Improve Equitable Access to Self‐Collection
Source: Cancer Med. 2025 May 25;14(11):e70981. doi: 10.1002/cam4.70981 (PMC12104203; doi:10.1002/cam4.70981)
Supplement: Supplementary file 1 — Data S1. [file CAM4-14-e70981-s001.docx]

**Supplementary material**

“I don’t think there is a one-size-fits-all”: A qualitative study exploring healthcare professional and service provider perspectives of using innovative models of cervical screening to improve equitable access to HPV self-collection

Bavor, C et al.

1. **COREQ Checklist**

| **Item number** | **Guide questions/description** | **Reported on Page #** | **Comment** |
| --- | --- | --- | --- |
| **Domain 1: Research team and reﬂexivity** | | | |
| *Personal Characteristics* | | | |
| 1. Interviewer/facilitator | Which author/s conducted the interview or focus group? | 8 | - Claire Bavor - Tessa Saunders - Mikayla Wolfe - Nicola Creagh - Claire Nightingale - Madeleine Clarke, Kate Flynn, Santhiya R (Acknowledgements) |
| 2. Credentials | What were the researcher’s credentials? e.g. PhD, MD | N/A | All authors have a bachelor’s degree, masters degree and/or a PhD. |
| 3. Occupation | What was their occupation at the time of the study? | N/A | All authors, expect one, were employed in a research capacity at the time of the study across various research institutes/organisations. One author was a Master of Public Health student. |
| 4. Gender | Was the researcher male or female? | 8 | All authors are women. |
| 5. Experience and training | What experience or training did the researcher have? | 8, 9 | All authors have received university-level training in qualitative data collection and analysis. |
| *Relationship with participants* | | | |
| 6. Relationship established | Was a relationship established prior to study commencement? | N/A. | Relationships between participants and researchers were not established for the purpose of this study. As the researchers used their network to recruit participants, some participants had a professional relationship with the research team. |
| 7. Participant knowledge of the interviewer | What did the participants know about the researcher? e.g. personal goals, reasons for doing the research | N/A. | Participants received a plain language statement informing them about the study and the research team. |
| 8. Interviewer characteristics | What characteristics were reported about the interviewer/facilitator? e.g. Bias, assumptions, reasons and interests in the research topic | N/A. | None. |

| **Domain 2: study design** | | | |
| --- | --- | --- | --- |
| *Theoretical framework* | | | |
| 9. Methodological orientation and Theory | What methodological orientation was stated to underpin the study? e.g. grounded theory, discourse analysis, ethnography, phenomenology, content analysis | 9 | The data was analysed thematically using template analysis. |
| *Participant selection* | | |  |
| 10. Sampling | How were participants selected? e.g. purposive, convenience, consecutive, snowball | 7 | Purposive and snowball recruitment methods were used. |
| 11. Method of approach | How were participants approached? e.g. face-to-face, telephone, mail, email | 7 | Email, social media, newsletters |
| 12. Sample size | How many participants were in the study? | 9 | There were 132 participants. |
| 13. Non-participation | How many people refused to participate or dropped out? Reasons? | N/A | The response rate is unknown due to the nature of recruitment. |
| *Setting* | | | |
| 14. Setting of data collection | Where was the data collected? e.g. home, clinic, workplace | 8 | Data was collected online, using Zoom. |
| 15. Presence of non-participants | Was anyone else present besides the participants and researchers? | N/A. | There were one or two members of the research team who were present for the interview only. |
| 16. Description of sample | What are the important characteristics of the sample? e.g. demographic data, date | 9, 10 | The gender, location, roles, role in cervical screening, and types of healthcare service of all participants is reported. |
| *Data collection* | | | |
| 17. Interview guide | Were questions, prompts, guides provided by the authors? Was it pilot tested? | Suppl. material | The types of questions included in the interview are provided in supplementary material. The interview guide was not piloted tested, reviewed by an Advisory Committee comprised of health service providers in clinical and non-clinical roles and Community and Consumer Advisory Panel comprised of women in the community. |
| 18. Repeat interviews | Were repeat interviews carried out? If yes, how many? | N/A. | Repeat interviews were not conducted. |
| 19. Audio/visual recording | Did the research use audio or visual recording to collect the data? | 8 | Interviews were audio recorded. |
| 20. Field notes | Were ﬁeld notes made during and/or after the interview or focus group? | N/A. | Yes. Notes were made by the interviewer during the interviews to aid with later analysis and context. |
| 21. Duration | What was the duration of the interviews or focus group? | 9 | The average time for an interview was 47 minutes (range 24-78 minutes). |
| 22. Data saturation | Was data saturation discussed? | N/A. | Purposive sampling was used to obtain a diverse sample of participants from across Australia rather than to obtain data saturation. |
| 23. Transcripts returned | Were transcripts returned to participants for comment and/or correction? | 8 | Participants could request a copy of their transcript to review it for accuracy and completeness. |
| **Domain 3: Analysis and ﬁndings** | | | |
| *Data analysis* | | | |
| 24. Number of data coders | How many data coders coded the data? | 9 | One author (CB) coded the data. Coding was reviewed and revised regularly with authors CN and TS. |
| 25. Description of the coding tree | Did authors provide a description of the coding tree? | Suppl. material | The coding framework has been supplied as supplementary material. |
| 26. Derivation of themes | Were themes identiﬁed in advance or derived from the data? | 9 | As template analysis was used, key themes were identified before coding commenced based on the research questions and the literature. Themes and codes were revised regularly throughout the coding process. |
| 27. Software | What software, if applicable, was used to manage the data? | 9 | NVivo (release 1.6.1, QSR International Pty Ltd). |
| 28. Participant checking | Did participants provide feedback on the ﬁndings? | N/A. | Participants did not provide feedback. |
| *Reporting* | | | |
| 29. Quotations presented | Were participant quotations presented to illustrate the themes/ﬁndings? Was each quotation identiﬁed? e.g. participant number | 10-15 | Quotes have been presented with the participants ID number, role and population group worked with. |
| 30. Data and ﬁndings consistent | Was there consistency between the data presented and the ﬁndings? | 10- | There was consistency between the data and findings. |
| 31. Clarity of major themes | Were major themes clearly presented in the ﬁndings? | 10-15 | Three overarching themes have been presented. Subthemes have been included. |
| 32. Clarity of minor themes | Is there a description of diverse cases or discussion of minor themes? | 10-15 | Differences in participants perspectives have been included. |

1. **Coding framework**

| **Models of screening** |  | **Theme** | **Subthemes** | |
| --- | --- | --- | --- | --- |
| Community events |  | Acceptability of innovative models of cervical screening | Acceptable |  |
| Community outreach |  |  | Not acceptable |  |
| Home in-reach |  |  | Role of the healthcare professional in screening |  |
| Mail-models |  | Appropriateness of innovative models of screening | Enablers to participation |  |
| Peer-supported services |  |  | Barriers to participation |  |
| Antenatal care |  | Maintaining cervical screening quality and safety | Information provision |  |
| Pharmacy pick-up |  |  | Establishing eligibility |  |
| Mobile screening services |  |  | Follow-up |  |
| Telehealth |  |  | Quality assurance |  |
| Innovative models in general |  |  | Privacy |  |
| Note. Each theme/subtheme was organised under each model of screening |  | Implementation considerations | Processes, roles and responsibilities in the cervical screening pathway | Governance |
|  |  |  |  | Logistics of providing and returning the self-collection kit |
|  |  |  |  | Pathology considerations |
|  |  |  | Scope of practice for nurses, midwives and other non-clinical providers |  |
|  |  |  | Adequate funding |  |
|  |  |  | Partnerships |  |
|  |  |  | Professional development |  |
|  |  |  | Co-design |  |
|  |  |  | Adequate staffing |  |
|  |  |  |  |  |

1. **Interview guides**

**Overview of semi-structured interview guides developed for each participant group and questions from the interview guide as relevant to this study**

| Guide | Population group served | Study participant role | Service type | Questions from interview guide |
| --- | --- | --- | --- | --- |
| 1 | General population | Clinical: Healthcare professionals | Primary and tertiary health services (e.g. primary healthcare services, sexual and reproductive healthcare services, public hospitals) | (A list of innovative screening models* were presented to the participant, which often prompted discussion about their acceptability and appropriateness)   1. For the screening models that you are particularly interested in, what do you think the implications would be on screening participation, safety, and quality if they were implemented? 2. For cervical screening providers:    1. How would you feel about overseeing or training non-clinical providers to deliver any of the models of screening listed? 3. Are there any other models of screening that you have implemented or think could improve access and participation in cervical screening? 4. What has been/what could be implemented to facilitate the implementation of innovative models? |
| 2 | Women and people with a cervix who are from refugee and asylum seeker backgrounds, have a physical or sensory disability and/or identify as part of the LGBTQI+ community | Clinical: Healthcare professional  Non-clinical: Service providers | Health and community services | 1. Has your service implemented any flexible models of screening*? 2. For those who have implemented flexible models:    1. Can you tell me about how this has worked in your service?    2. How did you get this happening?    3. What does/did this look like? (who is/was involved, when did you start?)    4. Did you overcome any challenges to implement this model? Tell me about this.    5. What has the feedback from clients been like? 3. For those who have not implemented flexible models?    1. Do you think any flexible models would well in your service, for your clients? Why/why not? 4. Are there any other flexible models that could work well for your clients? |
| 3 | General population | Pathology providers (e.g. pathologists, laboratory managers) | Public and private pathology laboratories | 1. Would you be supportive of the following models of screening*? |

*List of innovative models included:

- Opt-in mail model- Eligible screening participants are invited to screen, but must opt-in to receive a self-collection pack in the mail
- Opt-out mail model- All eligible screening participants are sent a self-collection pack in the mail
- Telehealth- Self-collection is offered during a telehealth consult and the patient is sent the self-collection pack or collects it at a pick-up point
- Pharmacy pick-up- Eligible screening participants can pick up and return self-collection pack at the pharmacy
- Community events - Self-collection is offered at community events, for example, at community cultural events and music festivals
- Community outreach – self-collection is offered within communities outside of the health service’s usual setting.
- Peer-led services- Self-collection is offered at services led by peers for peers, for example, LGBTQ+ peer workers supporting self-collection at LGBTQ+ health clinics
